# The Role of Anti-Thymocyte Globulin or Alemtuzumab-Based Serotherapy in the Prophylaxis and Management of Graft-Versus-Host Disease

**DOI:** 10.3390/biomedicines5040067

**Published:** 2017-11-29

**Authors:** Robert Ali, Jeremy Ramdial, Sandra Algaze, Amer Beitinjaneh

**Affiliations:** 1 Hematology/Medical Oncology Fellow, University of Miami/Miller School of Medicine, Miami, FL 33136, USA; robert.ali@jhsmiami.org (R.A.); jeremy.ramdial@jhsmiami.org (J.R.); 2 Internal Medicine Residency Program, University of Miami/Miller School of Medicine, Miami, FL 33136, USA; sandra.algaze@jhsmiami.org; 3 Associate Professor of Medicine, Stem Cell Transplant and Cellular Therapy Program, Sylvester Comprehensive Cancer Center, University of Miami/Miller School of Medicine; Miami, FL 33136, USA

**Keywords:** GVHD, ATG, alemtuzumab, serotherapy, thymoglobulin, allogeneic

## Abstract

Allogeneic hematopoietic stem cell transplant is an established treatment modality for hematologic and non-hematologic diseases. However, it is associated with acute and long-term sequelae which can translate into mortality. Graft-versus-host disease (GVHD) remains a glaring obstacle, especially with the advent of reduced-intensity conditioning. Serotherapy capitalizes on antibodies which target T cells and other immune cells to mitigate this effect. This article focuses on the utility of two such agents: anti-thymocyte globulin (ATG) and alemtuzumab. ATG has demonstrated benefit in prophylaxis against GVHD, especially in the chronic presentation. However, there is limited impact of ATG on overall survival and it has little utility in the treatment context. There may be an initial improvement, particularly in skin manifestations, but no substantial benefit has been elicited. Alemtuzumab has shown benefit in both prophylaxis and treatment of GVHD, but at the consequence of a more profound immunosuppressive phase, mandating aggressive viral prophylaxis. There remains heterogeneity in the doses and regimens of the agents, with no standardized protocol in place. Furthermore, it seems that once steroid-refractory GVHD has been established, there is little that can be offered to offset the ultimately dismal outcome. Here we present a systematic overview of ATG- or alemtuzumab-based serotherapy in the prophylaxis and management of GVHD.

## 1. Introduction

Although allogeneic hematopoietic stem cell transplant (HSCT) remains a viable option for the management of multiple hematological and non-hematologic conditions, it is associated with inherent risks which can significantly impair quality of life, or even result in death [1,2]. Currently, only about one third of patients have an HLA-identical sibling donor. However, establishment of a network of volunteer donor registries facilitate identification of a suitable match for up to 70% of cases [3]. Nonetheless, results of transplantation from HLA-compatible unrelated donors remain inferior compared to those of sibling donors, owing largely to higher rates of graft failure, infection and graft-versus-host disease (GVHD) [4,5,6,7,8]. That being said, the rates of transplant-related mortality (TRM) have declined over the past decade, primarily as a result of improved conditioning regimen, and advances in supportive care and infection management [9]. GVHD, however, still remains a pronounced issue, especially with the advent of reduced intensity conditioning (RIC), which capitalizes on graft-versus-tumor (GVT) effect to optimize responses [10]. The conditioning regimens usually involve chemotherapy, irradiation, and/or serotherapy, also referred to as “in vivo T cell depletion” [11,12]. It usually involves administration of either a polyclonal immunoglobulin, anti-thymocyte globulin (ATG), or alemtuzumab, a monoclonal antibody (mAb) specific for CD52 [13,14].

Both ATG and alemtuzumab suppress the reaction of host T cells, thus enabling engraftment [15]. Furthermore, due to their long half-lives, these agents persist following transplantation and eliminate donor T cells. This reduces the risk for GVHD, but at the consequence of attenuating the GVT benefit [16,17]. Moreover, they hinder post-transplant lymphocyte recovery, thereby augmenting the opportunity for viral infections, especially cytomegalovirus (CMV) and Epstein Barr virus (EBV) reactivation [18,19]. This effect is more prolonged with alemtuzumab as a result of its longer half-life [20]. Although alemtuzumab increases the risk for CMV infection, it may in fact reduce the overall risk for EBV-induced post transplantation lymphoproliferative disease when compared to ATG, as it depletes both B- and T-cell populations [21,22,23].

This review will focus on the utility of ATG- and alemtuzumab-based serotherapy in the prophylaxis and management of GVHD.

Options for GVHD prophylaxis are diverse, and are combined with myeloablative regimens, however no current standard exists [24]. Despite the use of pre-transplant GVHD immunosuppressive prophylaxis or T-cell depletion, the incidence of chronic GVHD (cGVHD) has been reported to be 6–80%, with about 30% of cGVHD cases presenting without preceding acute GVHD (aGVHD) and mortality is estimated to be around 50% [25]. GVHD perpetuates itself when certain cellular signals and damage to the host tissues result in the release of inflammatory cytokines leading to accumulation and activation of the immune effector cells [26]. In particular, interferon gamma, inerlukein-2, and tumor necrosis factor alpha are released leading to TH1 cytotoxic polarization [27,28]. Therefore treatments with ATG and alemtuzumab, which target T cells, seem logical for prophylaxis and therapy.

Acute and chronic GVHD have historically been differentiated by the time from transplant, however more recent classification systems have emphasized differentiating acute and chronic GVHD based on associated clinical manifestations. The National Institute of Health (NIH) Consensus Conference has suggested that GVHD be categorized as classic acute GVHD in the setting of typical acute GVHD clinical manifestations occurring less than 100 days from transplant and persistent, recurrent, or late acute GVHD in patients with typical acute GVHD symptoms experienced later than 100 days post-transplant. In contrast, chronic GVHD is classified as classic chronic GVHD in the presence of at least one diagnostic sign or at least one distinctive sign confirmed by biopsy, other tests or radiography in the same or other organ and at the exclusion of other diagnoses. Cases exhibiting signs and symptoms of both acute and chronic GVHD are identified as overlap syndrome [29]. Chronic GVHD often arises during immunosuppression tapering [30].

First line treatment of GVHD is with high dose steroids, typically methylprednisolone 1–2 mg/kg daily for seven to fourteen days, followed by a slow oral taper [31]. No optimal duration of steroid therapy has been determined in the literature [32]. Response to primary therapy positively correlates with survival, and poor response to steroids is associated with poor prognosis and a reported mortality rate of 70–90% [33]. Deaths are primarily due to relapse, infection or organ failure. Approximately 50–60% of patients do not respond to steroid therapy and require secondary systemic therapy [34,35]. There is no current consensus for the definition of steroid-refractoriness and indications for second line therapy are not well defined especially when isolated to one organ [36,37]. According to the American Society of Blood and Marrow Transplantation, second line therapy may be warranted in patients with progressive manifestations of GVHD after 3 days of treatment, persistent grade III GVHD after 1 week, un-improving grade II GVHD after 2 weeks, or not tolerating high dose steroids [31].

ATG and alemtuzumab both suppress host immune cells as they are anti-lymphocyte antibodies. Anti-lymphocyte antibodies are either polyclonal (ATG) or monoclonal (alemtuzumab). Immunosuppression is thus achieved by acting on T-cell surface antigens and depleting CD4 lymphocytes [38].

Two main polyclonal antibodies currently used include thymoglobulin and ATGAM [39]. Thymoglobulin is generated in rabbits, and hence commonly referred to as rabbit anti-thymocyte globulin, or rATG [40,41,42]. There are two forms of rATG; rATG-Thymoglobulin and rATG-Fresenius. The immunogen for rATG-Thymoglobulin is human thymocytes, while the immunogen for rATG-Fresenius, is a Jurkat T-cell leukemia line. The preparations differ in potency and efficacy [33,34]. A third polyclonal antibody ATGAM is a purified gamma globulin solution obtained by immunization of horses (equine) with human thymocytes. Studies have shown rATG used for GVHD prophylaxis seems not to increase relapse [40].

However, use of ATG is associated with cytokine release syndrome, and potential for developing post-transplant lymphoproliferative disorder (PTLD). These associations may not apply when lower dosing regimens are used [42]. ATG’s mechanistic effects are multifaceted including recent findings that have been demonstrated in its immunomodulatory activity; T-cell depletion in blood and peripheral lymphoid tissues through complement-dependent lysis, T-cell activation and apoptosis, modulation of key cell surface molecules that mediate leukocyte/endothelium interactions, induction of apoptosis in B-cell lineages, interference with dendritic cell functional properties, and finally, induction of regulatory T and natural killer T cells [13].

Another method to reduce GVHD is the use of monoclonal antibodies (mAb). For several years the genetically engineered anti-lymphocyte mAb, alemtuzumab has been used in vitro and in vivo for control of GVHD and prevention of rejection following bone marrow transplantation [43,44,45]. The antibody, which is directed against CD52, is extremely efficient at the lysis of target cells in the presence of human complement, but limited in vivo by the rate of complement biosynthesis [22,46]. CD52 is one of the most abundant membrane glycoproteins on T and B lymphocytes and is also expressed on NK cells, monocytes, macrophages, dendritic cells, and eosinophilic granulocytes and to a much lesser extent on neutrophilic granulocytes [45]. CD52 was shown to be a co-stimulatory molecule to activate CD4^+^ regulatory T cells [47]. Alemtuzumab is also known to induce antibody-dependent cellular cytotoxicity (after the activation of NK cells and macrophages through their IgG fragment C receptor). It also acts by directly inducing apoptosis. Depletion of peripheral lymphocytes occurs within one hour from alemtuzumab administration. The antibody has been shown to bind to human Fc receptors of NK cells and macrophages and is very effective for cell lysis in vivo [46]. The overall mechanistic approach leads to simple opsonization of bone marrow T-cells for subsequent clearance [38].

The predecessor of alemtuzumab is known as CAMPATH-1 (Cambridge Pathology) [22]. The concentration of CAMPATH-1G or CAMPATH-1H (an older version) [48] required for 90% saturation of the CD52 antigen is approximately 10–20 mg/mL [39]. Little or no reactivity with colony-forming cells could be detected by complement-mediated lysis, which is one of the parameters that made this method desirable. The antibody also removes residual host lymphocytes. One problem associated with the use of CAMPATH-1G in vivo was a significant delay (by up to 7 days) in neutrophil engraftment [43]. Because alemtuzumab could remain in the blood at the lympholytic level up to 1–2 months after transplantation, immune reconstitution was substantially delayed, leading to a high incidence of viral infection and relapse [49]. Comparison between ATG and CAMPATH is presented in Table 1.

## 2. Role of Serotherapy in Prophylaxis for Graft-Versus-Host Disease

### 2.1. Anti-Thymocyte Globulin

Given the significant morbidity and mortality associated with GVHD, multiple efforts have been ongoing to optimize prophylaxis for these sequelae. In vivo T-cell depletion via ATG has been a topic of much debate in this context. Several studies have evaluated the appropriate preparation, optimal dose and sequencing of therapy to capitalize on the immunosuppressive effects without heightening the risk for relapse and infection.

Two early meta-analyses have confirmed the benefit of ATG in preventing GVHD. Theurich et al. appraised the value of ATG as prophylaxis following allogeneic stem cell or bone marrow transplantation, including six randomized control trials up to February 2012 [50]. The incidence of severe aGVHD (grade II–IV) was significantly lower in patients who received ATG, risk ratio (RR) 0.68, 95% CI 0.55–0.85, *p* = 0.009, and number needed to treat (NNT) 8. Moreover, the use of ATG seemed to confer a lower incidence of cGVHD for those studies that reported this outcome. The incidence of relapse and non-relapse mortality was not significantly different in the ATG arms compared to the non-ATG arms, RR 1.13; 95% CI 0.75 to 1.68, *p* = 0.56, and HR 0.82; 95% CI 0.55 to 1.24, *p* = 0.35, respectively. Whereas Ziakas et al. evaluated various prophylactic regimens, either as monotherapy or in combination, including 33 studies up to June 2014 [51]. Though cyclosporine A (CsA)/methotrexate (MTX) was the most popularly utilized regimen, ATG/CsA/MTX was one of the superior treatment protocols, with odds ratios (OR) 0.45 (95% CI, 0.27–0.70), and NNT of 5. These two studies highlight the benefit of ATG in mitigating both acute and chronic GVHD, without adversely affecting mortality.

Optimizing GVHD prophylaxis among HLA-mismatched unrelated donors has also been a challenging dilemma. Ayuk et al. investigated the utility of ATG conditioning between 195 recipients of matched grafts and 64 recipients of mismatched grafts [52]. Patients received standard myeloablative conditioning, including ATG-Fresenius plus a combination of CsA and MTX as GVHD prophylaxis. ATG was dosed at 30 mg/kg/day within the last 3 days prior to transplant. Inclusion of ATG in the conditioning regimen led to a decreased incidence of both acute and chronic GVHD, as well as TRM, without compromising overall survival (OS) or disease free survival (DFS) (Table 2). The incidence of aGVHD among mismatched recipients was in fact noted to be even lower than that observed in the matched group. This may in part be due to the higher ATG dose administered in the mismatch group. Eighty five percent of patients in the mismatched group received a total ATG dose of 90 mg/kg, while only 40% of those in the matched group received this target. There were theoretical concerns of limiting relapse rate and hastening immunological recovery among the HLA-matched recipients, which prompted the dose-reduction. Nonetheless, the data ultimately showed that the addition of ATG can augment GVHD prophylaxis, such that mismatched grafts can be given without an increased risk of TRM.

Chronic GVHD also tends to be more common following peripheral blood stem cell transplant. Given the transition toward sourcing stem cells from peripheral blood, greater efforts have been placed to prevent the incidence of cGVHD [53]. Wolschke et al. investigated the impact ATG-Fresenius in HLA-identical or -mismatched sibling peripheral blood stem cell transplant [54]. The median dose was 30 mg/kg (range 20–90 mg/kg), in three divided doses administered daily, for 3 days prior to transplant. Fifty sixty percent (56%) of patients underwent standard myeloablative conditioning, with the remainder receiving RIC. The incidence of both aGVHD (II-IV) and cGVHD were lower in the ATG arm, but more notable was the difference in extensive GVHD at one year: 14% in the ATG arm compared to 39% in the non-ATG arm (*p* < 0.001). There was an increased incidence of EBV/post-transplant lymphoproliferative disease (PTLD) in the ATG-arm, 9% versus 2% (*p* = 0.05), however all patients could be salvaged with rituximab and no patient died due to EBV/PTLD. The rate of CMV reactivation was not statistically significant. The 5-year rate of relapse was 34% in the ATG arm, and 29% in the non-ATG arm, but this was not statistically significant (*p* = 0.3). The 5-year PFS, NRM and OS were 37%, 20% and 54% in the ATG arm, and 26%, 34% and 46% in the non-ATG arm, respectively.

Socie et al. also specifically addressed the role of ATG in preventing cGVHD [55]. Two hundred and one patients underwent myeloablative conditioning before transplantation from either matched or mismatched unrelated donors. ATG-Fresenius was administered at 20 mg/kg/day for three consecutive days prior to transplantation. The 3-year cumulative incidence of extensive cGVHD was 12.2% in the ATG group versus 45.0% in the control arm (*p* < 0.0001). The incidence of relapse and non-relapse mortality was 32.6% and 19.4% in the ATG arm, and 28.2% and 33.5% in the control arm, respectively. Three-year OS was 55.2% in the ATG group and 43.3% in the control group. Furthermore, the 3-year probability of survival, free of immunosuppressive therapy, was 52.9% in the ATG arm, and 16.9% in the non-ATG arm.

This benefit was further corroborated by Kroger et al. who conducted a prospective, multicenter, open-label, randomized phase 3 study employing ATG prophylaxis as part of the conditioning regimen [56]. One hundred and sixty eight patients were enrolled and assigned a 1:1 ratio for prophylaxis with ATG (10 mg/kg/day starting 3 days prior to transplant) versus without ATG. Patients received standard myeloablative therapy and transplantation of allogeneic peripheral-blood stem cells from an HLA-identical donor. Participants were followed for a median of 24 months. The cumulative incidence of cGVHD was 32.2% in the ATG arm, and 68.7% in the non-ATG arm. Two-year relapse free survival and OS rates were comparable in both arms: 59.4% and 74.1%, respectively, in the ATG group, and 64.6% and 77.9%, respectively in the non-ATG group. The rate of GVHD-free and relapse-free survival at 2 years was significantly higher in the ATG group than the non-ATG group (36.6% vs. 16.8%, *p* = 0.005). There was no significant difference for infectious complications or adverse events between groups.

There has been much ambiguity with regards to the optimal dosing of ATG for GVHD prophylaxis. Bryant et al. compared low dose ATG for prophylaxis of GVHD in MUD allogeneic HSCT, using rATG-Thymoglobulin at 0.5 mg/kg on day −2, and 2 mg/kg on day −1 pre-transplant [57]. Seventy two percent (72%) of patients received standard myeloablative conditioning, and the remaining had RIC. ATG exposed patients had lower rates of GVHD compared to the unexposed group (57% vs. 79%, *p* = 0.005), with the difference more pronounced in cGVHD (18% vs. 44%, *p* = 0.009). At median follow-up of 13 months for the ATG group and 20 months for the non-ATG group, there was no difference in OS (median OS not met for either cohort) or cumulative incidence of relapse (26% vs. 29%; *p* = 0.73). Two-year GVHD-free Relapse-free Survival (GRFS) between ATG exposed and unexposed cohorts was 23% vs. 3% respectively (*p* = 0.003). These findings indicate that lower doses of ATG can prevent GVHD, especially cGVHD, while still maintaining comparable survival outcomes.

Reduced-dosing options were also investigated by Tandra et al., who employed the use of rATG 3 mg/kg on days −2 and −1 pre HSCT among 58 MUD transplant patients [58]. Thirty-one (31) patients had RIC, and 26 were ablative. The 100-day incidence of grade III to IV aGVHD was 18%, and for cGVHD at 1-year it was 27%. The incidence of CMV, EBV and HHV6 viremia was 25%, 35% and 14%, respectively. Three-year overall survival estimate was 48%. Incidence of non-relapse mortality and relapse at 1 year was 21% and 44%, respectively. Furthermore, there was no report of EBV-driven lymphoproliferative disorder. This study also emphasizes the benefit of lower dose of ATG in reducing the rates of cGVHD.

Crocchiolo et al. compared rATG Thymoglobulin at 2.5 mg/kg on day −3, versus 2.5 mg/kg on days −2 and −1 prior to transplantation (HLA matched or mismatched donors) with the standard conditioning of busulfan and fludarabine [59]. The two-day regimen demonstrated improved rates of aGVHD and cGVHD: 23% versus 42% (*p* = 0.002), and 35% versus 69% (*p* < 0.0001), respectively. Furthermore, no statistical difference was observed with 2-year OS or NRM between arms, and the cumulative incidences were nearly identical (67% versus 65% and 19% versus 20%, respectively). The incidence of relapse/progression was lower in the two-day arm, 19% compared to 30%. These data suggest that ATG at a total dose of 5 mg/kg correlates significantly with reduced incidence and severity of GVHD without compromising disease control. This alludes to a step forward in the optimization of RIC.

As a further step, Wang et al. investigated the influence of two different doses of ATG on the incidence of GVHD among patients receiving HSCT from haploidentical donors without ex vivo T-cell depletion [60]. Patients underwent myeloablative conditioning. The dosing schedules were as follows: ATG (Thymoglobulin; rATG) 2.5 mg/kg/day (cumulative dose 10 mg/kg, ATG-10) or 1.5 mg/kg/day (cumulative dose 6 mg/kg, ATG-6) on days −5 to −2 prior to transplant. The incidence of aGVHD (III–IV) was higher in the ATG-6 arm (16.1%), than in the ATG-10 arm (4.5%, *p* = 0.005). Also, while there were no deaths attributed to aGVHD in the ATG-10 arm, the incidence was 4.5% in the ATG-6 group. Conversely, the rate of EBV reactivation was higher in the ATG-10 group: 25.3% versus 9.6% (*p* = 0.001), with a corresponding higher rate of PTLD: 8% versus 1.8%, respectively. The 1-year incidence of CMV reactivation was comparable in both groups. Though no significant difference in septicemia or invasive fungal infections was shown between both arms, there was a trend toward higher incidence of these in the ATG-10 group (11.6% versus 5.4%, respectively). The 1-year probability of NRM, OS and DFS also did not differ. While the higher dose ATG may have reduced the incidence of aGVHD and death secondary to aGVHD complications, this advantage did not translate into lower NRM or OS, likely as a result of delayed immune reconstitution and increased susceptibility to infections. Likewise, the higher rate of severe GVHD observed in the 6 mg/kg arm offset the decreased mortality from non-GVHD complications, thus also resulting in similar survival outcomes.

Additionally, the long term benefits of ATG were gauged in an updated study by Bacigalupo, et al. [61]. The sample size included 100 participants who underwent bone marrow transplant from unrelated donors. They were randomized to receive rATG total dose 7.5 mg/kg versus no ATG. All patients were prepared with conventional cyclophosphamide and total body irradiation, followed by cyclosporine/methotrexate as GVHD prophylaxis. Median follow-up was 5.7 years. When comparing the ATG arm to the non-ATG arm, the rate for cGVHD was 37% versus 60%, respectively, and for extensive cGVHD it was 15% versus 41%, respectively. Chronic lung dysfunction was diagnosed in 19% of patients, compared to 51%; and Karnofsky performance status >90% at 4 years was 89% in the ATG arm compared to 57% in the control arm. For patients who survived 1 year, TRM was 3% in the ATG arm versus 25% in the non-ATG arm, and the survival was 85% versus 58%, respectively. The 6-year survival between ATG versus non-ATG patients was 31% and 44%, respectively (*p* = 0.080). Rate of relapse was higher among the ATG patients (40%) versus the control arm (32%), *p* = 0.09. Overall, the addition of ATG to standard CsA/MTX prophylaxis improved extensive cGVHD, chronic lung dysfunction, as well as quality of life.

Ultimately, there still exists much variability in standard dosing for ATG prophylaxis. Thymoglobulin is typically administered at 2.5 to 10 mg/kg, with the European Blood and Marrow Transplant Group recommending a total dose of 7.5 mg/kg, divided into three days for MUD transplants. In the United States however, different protocols use different doses of rATG, with the most common total dose being 4 mg/kg administered in divided doses over the course, divided into three days [62].

In an attempt to answer the question of optimal dosing of ATG from another perspective, Admiraal et al. looked at the relationship between prophylactic exposure to ATG and clinical outcomes in adult patients with acute leukemia and myelodysplastic syndrome. Those patients who received unrelated stem cell transplant and fell into what they deemed the optimum exposure group had a greater chance of event-free survival than those in the below optimum exposure group. Interestingly, the “optimum exposure” was based on absolute lymphocyte counts rather than weight-based-dosing [63]. This then brings to question whether therapies acting on the immune system should be dosed based on other metrics than just weight.

Finally, there have been a few other studies addressing the benefit of different ATG preparations. The general consensus was that for the prophylaxis of GVHD, rabbit ATG has proven to be efficacious, whereas equine ATG has not demonstrated any notable benefit [64,65,66].

### 2.2. Alemtuzumab

Alemtuzumab-conditioning regimens consistently confer an advantage in the reduction of acute and cGVHD, and by extension TRM [10,67,68]. Furthermore, with the convention toward RIC regimen to exploit the benefit of GVT effect, the incidence of grade II-IV aGVHD averages 40–60%. Alemtuzumab overcomes this drawback by reducing this incidence to 10–20% [10,68,69].

A pioneer phase II study performed by van Besien, et al. (2005) sought to evaluate the benefit and toxicity of including alemtuzumab with fludarabine/melphalan conditioning regimen for AML and MDS [70]. Donors were either related or unrelated with no more than one antigen mismatch after considering HLA-A, B, C and DR. The study enrolled 52 adult patients, averaging 52 years of age, and with the majority having high-risk disease, comorbidities and/or modest reduction in performance status. The cumulative probability of grade II to IV aGVHD was 33% at 1 year, and 18% for cGVHD at 1 year. All five patients with extensive cGVHD were recipients of unrelated donor transplants. Compared to their prior studies, sans alemtuzumab, the rate of GVHD was dramatically reduced. Furthermore, they employed aggressive CMV prophylaxis protocols to attenuate CMV reactivation. At one-year, OS was 48%, PFS was 38% and TRM was 33%.

Given these promising results, this group then performed a follow-up study (2009), comparing outcomes of 95 patients treated at the University of Chicago with fludarabine/melphalan/alemtuzumab conditioning to 59 patients treated at M.D. Anderson Cancer Center with only fludarabine/melphalan conditioning [71]. Patients were diagnosed with AML or MDS, and had similar patient and donor characteristics. The rate of aGVHD was 23.3% among patients conditioned with alemtuzumab, versus 58.1% in the non-alemtuzumab group. More strikingly, the rate of cGVHD was reduced almost 5-fold, with an incidence of 16% among those treated with the alemtuzumab-containing regimen. Additionally, there was a non-significant increase in recurrence rates (24%) with alemtuzumab, and a non-significant decrease in TRM (25%), but with nearly identical OS between both groups.

A parallel study was performed by Malladi, et al. who retrospectively analyzed 88 patients, assessing the effect of in vivo T-cell depletion in HLA-identical sibling RIC allografts for adult AML by comparing patients who received alemtuzumab (*n* = 51) to those without alemtuzumab (*n* = 37) conditioning [72]. Results mirrored those of the van Besien study, with minor decrease in TRM, increase in relapse rates and similar OS rate in the alemtuzumab group. Moreover, despite only a modest decline in grades II-IV aGVHD (14% vs. 22%), the reduction in the rate of cGVHD was more profound, meeting statistical significance (23% vs. 77%, *p* = 0.001, Table 3).

Alemtuzumab has also demonstrated utility when incorporated into RIC regimen used to transplant aggressive non-Hodgkin’s Lymphoma (NHL). Such patients have either refractory disease or relapse following an autologous transplant, and myeloablative regimen are associated with a high rate of TRM [73]. Morris and Mackinnon evaluated the outcome for relapsed and refractory NHL treated with alemtuzumab containing RIC. Eight eighty patients were studied, with the majority diagnosed with low- or high-grade lymphoma, and 10 patients with mantle cell lymphoma. GVHD prophylaxis was with CsA. 15% of patients developed grade II-IV aGVHD and the incidence of cGVHD was 6.8%. The addition of alemtuzumab to RIC has resulted in lower rates of grade II-IV aGVHD (10–17%) and cGVHD (7–18%), without compromising OS [74,75]. The 100-day and 3-year non-relapse mortality for all patients was 14% and 22% respectively [75], showing a role for alemtuzumab added to RIC.

Cons of alemtuzumab include an association with a protracted immunosuppression phase, with very slow recovery of CD4^+^ cells, predisposing patients to opportunistic infections [69,70]. CMV reactivation is among the more common infections, with an incidence ranging 30% to 85% [10,70,74,75,76,77]. With the prompt institution of anti-viral therapy, namely ganciclovir or valganciclovir, most CMV reactions can be well-contained [78]. There is also an increased risk of EBV infection, but given the concomitant depletion of B cells and anticipatory administration of rituximab, the risk of developing a post-transplant lymphoproliferative disorder is low [79].

Other caveats of alemtuzumab conditioning are the risk for disease recurrence following SCT and mixed T-cell chimerism [2]. The T-cell depletion inherent to alemtuzumab is thought to predispose to disease recurrence after transplant [80]. This can be overcome with further chemotherapy, tyrosine kinase inhibitors in CML, and/or donor lymphocyte infusion (DLI), thus maintaining a respectable OS [2]. The value of converting to full donor chimerism remains equivocal in obtaining long-term disease control, with speculation that this may decrease relapse and facilitate prolonged disease control.

## 3. Role of Serotherapy in the Treatment of Graft-versus-Host Disease

### 3.1. Anti-Thmocyte Globulin

Acute GVHD is one of the major obstacles for successful HSCT, with mortality rates for severe aGVHD (grades III–IV) ranging 70–100% [81,82,83]. As such, Cragg et al. assessed the role for upfront ATG treatment in a randomized trial comparing prednisone with ATG/prednisone as initial systemic therapy for moderately severe aGVHD [84]. Forty six patients were randomly assigned to therapy with prednisone (60 mg/m^2^/day for 7 days), and 50 patients were treated with ATG/prednisone (15 mg/kg/twice daily ATG, plus 20 mg/m^2^/twice daily prednisone for 5 days). Each arm was succeeded by an 8 week prednisone taper. The overall response at day 42 was equivalent in both arms, 76% (*p* > 0.8). Furthermore, complications were more frequent in the ATG/prednisone group: a greater number CMV infections (44% versus 22%, *p* = 0.2) and more frequent pneumonitis (50% versus 24%, *p* < 0.1). EBV lymphoproliferative disease was uncommon (4 cases), and comparable in both groups. There was no significant difference in survival at up to 2 years between both groups. ATG/prednisone failed to improve control of GVHD, while potentially affecting survival secondary to infectious complications. Consequently, combination therapy with ATG should be reserved as second-line therapy in the management of aGVHD.

Thereafter, Remberger et al. investigated ATG in the context of severe steroid-resistant acute GVHD [85]. Patients with grade II-IV aGVHD involving the skin, liver and gastrointestinal tract were included in the treatment arm (*n* = 29). Five different preparations of ATG were used, with corresponding doses: Rabbit ATG (0.25–1 mg/kg/day), BMA 031 (0.2 mg/kg/day), OKT-3 (2.5–5 mg/day), rATG-Fresenius (4 mg/kg/day), rATG (2 mg/kg/day). The control group consisted of all other patients who developed aGVHD grade III-IV, but did not receive ATG treatment (*n* = 33). Corticosteroids formed the basic GVHD treatment backbone, with additional therapies such as PUVA, MTX, thalidomide, extra-corporeal PUVA, CsA, tacrolimus and MMF also being employed.

The rate of response was most pronounced among those patients with skin involvement (72%). Nonetheless, survival at 100 days was 37% and at 1 year was 12%, with most patients succumbing to GVHD (72% aGVHD, 14% cGVHD). Viral and fungal infections also contributed to mortality. Results from the control group paralleled that of the ATG-treated group, with similar survival rates among patients of high-grade GVHD. Furthermore, the cause of death was also similar between groups, being mainly attributed to GVHD, and then infectious etiology. Ultimately, this study highlighted that the addition of ATG to treat severe aGVHD offered no improvement when compared to those patients who were managed without ATG. This postulates that once severe GVHD is established, treatment is difficult, regardless of intervention. It also challenges whether the treatment paradigm should focus on starting therapy upfront for patients with grade I disease.

In terms of response and OS, Ozen et al. corroborated these findings on their 20-year retrospective study evaluating the use of ATG for the treatment of steroid-refractory aGVHD [86]. Patients were diagnosed with severe (grade III-IV) aGVHD with involvement of the skin, liver or GI tract. Three different preparations of ATG were utilized: Fresenius, Thymoglobulin and Lymphoglobulin, at an average dose of 2–10 mg/kg/day for 5 days. An overall response was noted in 42% of patients, however the OS did not increase in any treatment group. High mortality rates were largely due to infectious complications, occurring in 10 of the 15 responding patients, and 19 of the 20 non-responders (Table 4).

Other studies assessing the merit of ATG in this setting have yielded similar discouraging results. Khoury et al. observed an initial 30% improvement in patients treated with ATG for steroid refractory aGVHD [87]. Despite this, 52 patients (90%) died at a median of 40 days after ATG therapy, with 74% of cases being attributed to progression of aGVHD and/or infection. ATG dosing was variable from 10 to 40 mg/kg/day, for up to 10 days in total. Arai et al. also demonstrated an overall response of 30%, with the most distinct benefit among those patients with skin disease (59%) [88]. Nonetheless, median OS was 3.6 months, with 10% of patients alive at 2 years. Only 5% of the deaths were due to relapse, with the remaining causes secondary to GVHD, infection and/or organ failure. The dose of ATG was 10–15 mg/kg on alternate days for 7 treatments, repeating if there was a response.

The influence of varied ATG doses was also evaluated by McCaul et al. [89]. Thirty six patients with grades II-IV steroid-resistant aGVHD were treated with rATG according to either of the two following regimens: 2.5 mg/kg/day × 4–6 consecutive doses, or 2.5 mg/kg/day on days 1, 3, 5, 7. The overall response rate (ORR) was 59%. However, similar to Khoury et al., OS rates were poor. Only 2 patients (6%) were alive at 15-plus and 34-plus months, with opportunistic infections, as well as development of PTLD (25%) contributing to mortality.

Additionally, MacMillan et al. retrospectively assessed the benefit of early ATG therapy in 79 patients with steroid-resistant aGVHD [90]. Patients were treated with Equine ATG at a dose of 15 mg/kg/twice daily for 5 days, for up to 5 courses (median of 2 courses). Time to initiation of ATG was 16 days (median, 5 to 44 days) after the onset of GVHD. ORR was 54%, with the best responders among those with skin disease (61%). Patients in whom ATG therapy was initiated within 2 weeks of steroid treatment had a higher probability of survival than did patients with a longer period between treatment regimen, 46% versus 19%, respectively (*p* = 0.05). The estimated survival at 1 year was 32%. The primary cause of death was GVHD in 48 patients (89%) and relapse in 6 patients (11%), with infections contributing to 36 deaths (67%). These data indicate that though OS is generally poor for this group of patients, at that the impact of ATG may be more marked if initiated earlier.

Given that several trials have demonstrated consistently poor outcomes with the use of ATG for steroid refractory aGVHD, Hsu et al. sought to explore the global perspective on this subject [91]. An international survey was conducted with responses from 153 centers in 36 countries. Overall, 67% of the reporting centers indicated the routine use of ATG to manage steroid-refractory GVHD, with a higher proportion of centers being pediatric-oriented. Equine ATG was used among 50% of centers and rATG by 24% of centers. There was considerable variability in the dose of ATG employed: Equine ATG–averaged from 5 mg/kg to 40 mg/kg, with a minimum cumulative dose 25 mg/kg to 180 mg/kg; rATG—0.5 mg/kg to at least 10 mg/kg, with a minimum cumulative dose of 4 mg/kg to 50 mg/kg. Based on this review, there is no uniform consensus on the indication of ATG for the management of steroid refractory aGVHD, nor is there standardization of the appropriate preparation or dosing that should be recommended.

Presently, there is no uniform consensus on the indication of ATG for the management of steroid refractory severe aGVHD. An initial response is often observed, particularly among those with skin manifestations, however, this benefit is offset by infectious complications and ultimate progression of GVHD. ATG has not demonstrated a consistent benefit in overall survival when employed in this context. Furthermore, there is no standardization on the appropriate preparation or dosing that should be recommended. Nonetheless, ATG should be considered for the management of steroid refractory aGVHD, as it seems to offer a greater benefit when started within 2 weeks of steroid initiation.

### 3.2. Alemtuzumab

The pathophysiology of aGVHD hinges on the presentation of host antigens to donor T cells by antigen presenting cells, such as dendritic cells [91,92,93,94]. Given that alemtuzumab depletes T cells and dendritic cells from both the donor and recipient, it may be beneficial for not just preventing, but also for managing aGVHD [18,69,95,96,97].

This concept was explored by Gomez-Almaguer et al., who evaluated the safety and efficacy of alemtuzumab in treating steroid-refractory aGVHD, grade II and higher, among 18 patients [98]. Over ninety percent of these patients had HLA-identical sibling donors, and received RIC. Alemtuzumab was scheduled at 10 mg/day for 5 consecutive days, with response being assessed by day 28. The ORR was 83%, with 6/18 patients achieving a complete response. Ten of the 15 responders are alive at median follow-up of 11 months. The three patients who did not respond died from GVHD. CMV reactivation occurred in 11 patients (61%).

Martinez et al. also conducted a similar trial, enrolling 10 patients with grade III-IV steroid-refractory aGVHD [99]. They were also able to demonstrate a reasonable overall response, with 2 patients (20%) experiencing a complete response and 3 patients (30%) undergoing a partial response. Conversely, their survival data was markedly different, with all 10 patients dying at a median of 40 days from alemtuzumab administration. Seven patients experienced progression of aGVHD, 2 succumbed to severe infection and 1 patient had a relapse of leukemia. Reactivation of CMV was noted among 7 patients. The difference in survival may be in part that Gomez-Almaguer enrolled patients with lower grade disease, whereas the participants for Martinez et al. were all grades III-IV. Nonetheless, these data demonstrate that steroid-refractory aGVHD may respond to alemtuzumab upfront, with a more durable response if introduced at a lower grade of disease. However, for those with established severe aGVHD, the advantage conferred by alemtuzumab does not offset the overall dismal outcome of this complication.

Two other studies also sought to ascertain the role of alemtuzumab in patients with disease confined to the gastrointestinal tract and/or liver. Schnitzler et al. evaluated 20 patients with histologically confirmed steroid refractory grade III-IV intestinal GVHD after related and unrelated HCT [100]. They demonstrated an overall response rate of 70%, with 8 patients achieving a complete response. The median time to clinical response was 12 days from the first alemtuzumab dose. They also demonstrated a median survival of 280 days and 1-year OS of 50%, which was superior, or at least on par, with other standard regimens. Infectious complications encountered were mainly *Aspergillus* pneumonia, bacterial pneumonia, CMV colitis and EBV reactivation. CMV reactivation was managed with ganciclovir and EBV reactivation with rituximab. Schub et al. also assessed 18 patients with grade III-IV disease confined to the gut and/or liver [100]. The dose of alemtuzumab varied among three sub-groups of patients, with a gradual downward titration from the original dose of 70–80 mg, to a final dose of 3–13 mg. This was done in an attempt to offset the higher frequency of virus reactivation and bacterial infections encountered with the higher dose alemtuzumab. Results were similar, with 17 patients (85%) responding to therapy, and 5 patients experiencing a complete response. Furthermore, one-third of patients were still alive at a median follow-up of 108 weeks (Table 5).

In this subgroup of patients diagnosed with gastrointestinal tract and/liver aGVHD, alemtuzumab clearly has demonstrable benefit, with well over two-thirds of the patients experiencing an overall response. However, with the profound T-cell depletion, viral reactivation and fungal and bacterial infections continue to be an obstacle. Aggressive surveillance and treatment strategies may prove efficacious in mitigating these sequelae. Nonetheless, overall survival rates were reasonable, alluding to a potential role of alemtuzumab in this setting. Further studies evaluating head-to-head comparison of dosing regimens will be helpful to elucidate a dose that limits infectious complications, without compromising clinical efficacy.

There has also been anecdotal evidence suggesting the role of alemtuzumab to manage cGVHD [101,102,103]. Ruiz-Arguelles, et al. described a case of a 19 year old female diagnosed with M2 acute myelogenous leukemia who underwent HLA-matched sibling allograft, and subsequently developed extensive, ulcerated, refractory cutaneous GVHD. She was treated unsuccessfully for 9 months with steroids, cyclosporine-A, sirolimus, tacrolimus, mycophenolate mofetil, infliximab and rituximab. Twenty one months following the allograft, she was treated with alemtuzumab 10 mg daily subcutaneously for 6 consecutive days every 4 weeks. Seven months following initiation of alemtuzumab, she experienced resolution of 100% of the ulcers, as well as the pain. The dose was then reduced to 10 mg every 10 days.

The patient was well for 20 months thereafter, however, she developed exertional dyspnea and severe hypoxemia after alemtuzumab was interrupted for a dental procedure. Chest X-ray showed diffuse reticulonodular pattern of disease. The patient declined pulmonary biopsy. She had no improvement with steroids, antibiotics or anti-viral agents. Alemtuzumab was re-started at 10 mg/day subcutaneously, and her respiratory symptoms and X-ray findings improved dramatically within 7 days. Though there was no histological confirmation of cGVHD, the clinical presentation and response to alemtuzumab are highly suggestive of cGVHD. More so, this reflects the potential role for alemtuzumab in the management of cGVHD.

Though a solitary case, the above report illustrates the potential for alemtuzumab in the management of cGVHD. It may be a worthwhile option when cGVHD is refractory to other modalities of therapy, and may even be considered earlier in the treatment to alleviate the steroid burden. Further investigation is mandated to ascertain the optimal initial dose, maintenance regimen and duration of therapy.

## 4. Conclusions

Rabbit ATG (and not equine) prophylaxis definitely reduces GVHD, primarily chronic and severe acute disease, thus ATG and its role in prophylaxis will improve quality of life. This effect does not seem to be associated with increased relapse or fatal infections, except for EBV. The impact of ATG will be better defined by the multi-center trial comparing ATG-F to no ATG (ClinicalTrials.gov identifier NCT01295710), which is completed and pending publication. Lower doses of ATG seem to prevent GVHD without increasing the risk of complications.

In terms of GVHD therapy, both equine and rabbit ATG show activity (about 30%), with a predilection for skin GVHD, and some reports even suggest better outcomes if initiated early. Regardless, most patients still die from either GVHD or infection within 100 days post-ATG [103]. On the other hand, based on small studies, alemtuzumab seems to have favorable efficacy on intestinal and liver GVHD with less risk of TRM. Randomized trials to compare the two serotherapies should be considered. It is of our group’s opinion that both ATG and alemtuzumab have their role in treating GVHD however optimal dosing strategies and timing of initiation of therapy still require further investigational studies.

In terms of prevention, alemtuzumab seems to be less of a desirable drug given the increased risk of fatal infections and possible relapse. In this setting, the beneficial effect of alemtuzumab seems to be limited to cGVHD and not aGVHD. Regarding ATG for prophylaxis it has been clearly demonstrated to have benefit in severe aGVHD. Dosing however still needs to be optimized and further evaluation of weight based dosing versus dosing based on absolute lymphocyte count should be pursued. Taking all of these variables into consideration it can be agreed, that despite several studies and retrospective analyses, there is still room for optimization of these two modalities as a part of the armamentarium that the modern transplanter can have at their disposal.

## Figures and Tables

**Table 1:** Comparing pharmacology of anti-thymocyte globulin (ATG) to that of CAMPATH.

Antilymphocyte Antibodies	rATG-Thymoglobulin	rATG-Fresenius	Campath-1G	Campath-1H
Type of antibody	Polyclonal	Polyclonal	Monoclonal	Monoclonal
Route of administration	IV	IV	IV/SubQ	IV/SubQ
Infusion reaction	Yes/CRS	Yes/CRS	Yes	Yes
Primary cells affected	CD4 lymphocytes	CD4 lymphocytes	T, B, Dendritic, NK, Monocyte, Macrophages	T, B, Dendritic, NK, Monocyte, Macrophages
Mean elimination T 1/2	29.8 days	29.8 days		
Mean overall T 1/2			12 days	8 days
CMV reactivation	+	+	+	+ *
EBV reactivation	++	+	+	+
Prolonged cytopenias	+	+	++	+
Delay in neutrophil engraftment	-	-	++	+
Secondary malignancy	++	+	-	-
Schedule	2–2.5 mg/kg daily × 3–5 days	2–2.5 mg/kg daily × 3–5 days	20 mg daily × 5 days	20 mg daily × 5 days

IV: Intravenous; SubQ: Subcutaneous; NK: Natural Killer cells; CMV: Cytomegalovirus; EBV: Epstein Barr virus. * Campath-1H is associated with earlier CMV reactivation. The designation of “+” indicates statistically significant occurrence, “-” indicates does not occur or not statistically significant occurrence, and “++” indicates that it occurs at a higher rate than the other comparators.

**Table 2:** Comparison of rates of GVHD, TRM, relapse and survival among HLA-matched and mismatched unrelated grafts receiving ATG.

	HLA-Matched (*n* = 194)	HLA-Mismached (*n* = 65)	*p*-Value
GRAFT FAILURE (%)	0.5	3	0.16
ACUTE GVHD (II-IV)	45	35	0.14
OVERALL CHRONIC GVHD	42	40	0.68
GVHD ASSOCIATED MORTALITY	7.7	6.2	-
TRM (AT 3 Years)	29	27	0.59
RELAPSE (AT 5 Years)	28	25	0.63
OS (AT 5 Years)	54	50	0.99
DFS (AT 5 Years)	43	47	1.0

GVHD: Graft-versus-host disease; TRM: Transplant-related mortality; OS: Overall survival; DFS: Disease-free survival.

**Table 3:** Comparison of studies performed by van Basien and Malladi, assessing the benefit adding alemtuzumab to standard conditioning [71,72].

	Van Basien			Malladi		
	Alemtuzumab-Containing Regimen	Without Alemtuzumab	*p*	Alemtuzumab-Contining Regimen	Without Alemtuzumab	*p*
Number	95	59		51	37	
Median age	54	55		51	51	
Conditioning	95	59				
Regimen						
Flu/Mel				39	20	
Flu/Bu				8	6	
Flu/Cy				2	8	
Other				3	2	
Dose Alemtuzumab	100 mg (20 mg/day, −7 to −3)			Average 60 mg (30–100 mg)		
TRM (%)	24.6 (1-year)	28.8 (1 year)	0.25	12 (2 year)	17 (2 year)	0.49
Relapse(%)	23.7 (1-year)	20.3 (1 year)	0.24	35 (2 year)	19 (2 year)	0.28
2 YR OS (%)	40.5	45.7	0.92	60	61	0.84
aGVHD (II-IV) (%)	8.6	16.5	0.08	14	22	0.25
cGVHD (%)	16	78.4	<0.01	23	77	0.001

Flu/Mel: Fludarabine/Melphalan; Flu/Bu: Fludarabine/Busulfan; Flu/Cy: Fludarabine/Cyclop hosphamide; TRM: Transplant-related mortality; OS: Overall survival; aGVHD: Acute graft-versus-host disease; cGVHD: Chronic graft-versus-host disease.

**Table 4:** Comparison among various trials on the use of ATG for the management of steroid refractory acute graft-versus-host-disease.

References	*n*	Grade	Sites	GVHD PPX	Type/Dose ATG	ORR (CR + PR)	Site of Best Response	Median Time to Onset of ATG	Infectious Complications	Survival	Cause of Death
Remberger et al., 2001 [85]	29	II–IV	Skin (93%) GI (76%) Liver (62%)	CsA/MTX—66% CsA + MTX—31% CsA + Steroid—3%	Rabbit ATG—0.25–1 mg/kg/day BMA 031—0.2 mg/kg/day OKT-3—2.5–5 mg/day Fresenius—4 mg/kg/day Thymoglobulin—2 mg/kg/day	49%	Skin—72%	Not stipulated	CMV reactivation—37% Fungal infection—31%	37%—at 100 day 12%—at 1 year 10%—after 3 year	GVHD—86% aGVHD—84% cGVHD—16%
Khoury et al., 2001 [87]	58	II–IV	Skin (74%) GI (81%) Liver (52%)	CsA—7% CsA + Prednisone—27% CsA/MTX/Prednisone—66%	Equine ATG 40 mg/kg/day × 4 day 15 mg/kg on alternate days × 6 days 10–20 mg/kg/day × 5–10 day	31%	Skin—79%	9 days after initiation of MP (3–39 day)	Incidence of all infections—67% Invasive deep fungal infection—51%	10%—at median 40 day	Infection ± GVHD—73% ARDS—15% Relapse—12%
Arai, et al., 2002 [88]	69	II–IV	Skin (81%) GI (59%) Liver (78%)	CsA—72% CsA + MTX—1% MTX—1% Other (CsA + elutriation or steroid)—25%	ATG 10–15 mg/kg on alternate days × 7 day, followed by 2nd dose if responding	30%	Skin—59%	46 day post-transplant, with median 24.5 day interval between primary and ATG salvage treatment	Viral, fungal infection–incidence not stipulated	10%—at 2 year	GVHD/infection/multi-organ failure—95% Relapse—5%
McCaul et al., 2000 [89]	36	II–IV	Skin, GI, Liver	CsA + short course MTX—81% CsA administered after T cell depletion of bone marrow—17% CsA/MTX/MP—3%	Rabbit ATG (Thymoglobulin) 2.5 mg/kg/day × 4–6 day, in succession 2.5 mg/kg/day on alternate days for 7 day	59%	Skin—96%	41 day post-transplant (16–204 day)	Systemic fungal infection—31% Viral infection—25% (CMV, disseminated VZV, influenza A pneumonia) Bacteremia—19%	6%—at 15+ and 34+ months	GVHD—28% Infection—28% PTLD—22% Relapse—11% Veno-Occlusive Disease—3% Intra-cranial Hemorrhage—3%
MacMillan et al., 2002 [90]	79	I–IV	Skin (81%) GI (80%) Liver (11%)	CsA/MTX—58% CsA/Prednisone ± other—9% MTX/ATG/Prednisone—13% Ex vivo T-lymphocyte depletion—13% Tacrolimus—35% MTX alone—5%	Equine ATG 15 mg/kg BID × 5 day, for 1–5 courses (median 2 courses)	54%	Skin—61%	After initiation of steroids—16 day After HSCT—48 day	Bacterial infection—37% Fungal infection—18% CMV infection—10% EBV Lymphoproliferative disease—1%	32%—at 1 year	GVHD—48% Relpase—11% Infections were contributing factor—67%
Ozen et al., 2015 [86]	35	III–IV	Skin (29%) GI (71%) Liver (25%)	CsA + short course MTX	Fresenius/Thymoglobulin/Lymphoglobulin 2–10 mg/kg/day × 5 day	42%	Not stipulated	15 day from diagnosis (7–10 day)	Bacterial and fungal infection—83%	No increase in any group	Responders: bacterial/fungal infection (10/15) Non-Responders: GVHD complications + Infection (19/20)

GVHD: Graft-versus-host disease; PPX: Prophylaxis; ORR: Overall response rate: CR: Complete response; PR: Partial response; CsA: Cyclosporine A; MTX: Methotrexate; PTLD: Post-transplant lymphoproliferative disease; MP: Methylprednisolone; CMV: Cytomegalovirus; EBV: Epstein-Barr virus.

**Table 5:** Comparison among various clinical trials on the use of alemtuzumab for the management of steroid refractory acute graft-versus-host-disease.

References	*n*	Dose	Median Time To Initiation	GVHD PPX	Site	GRADE	CR	ORR	Survival	CMV	Infectious Events
Gomez-Almaguer et al., 2008 [95]	18	10 mg/day SC × 5 days	Not stipulated	CsA + short course MTX	GI, Liver, Skin	≥2	33%	83%	55% (1 year)	61% (asymptomatic reactivation)	78%
Schnitzler et al., 2009 [97]	20	10 mg weekly	7 days	Not stipulated	GI	III–IV	40%	70%	50% (1 year)	10% (CMV Colitis)	Total events not specified
Martinez et al., 2009 [96]	11	10 mg/day IV × 5 days, followed by 10 mg/day weekly on Days 8, 15, 22 if CR not achieved	Not stipulated	CsA + MTX (myeloablative) CsA + MMF (fludarabine-based RIC)	GI, Liver, Skin	III–IV	20%	55%	0% (1 year)	64% (asymptomatic reactivation)	73%
Schub et al., 2011 [98]	18	70–80 mg, repeat after 3–4 weeks 20–33 mg every 2–3 weeks 3–13 mg every 2–3 weeks	33 days	CsA + MTX CsA + MMF	GI, Liver	III–IV	28%	94%	33% (108 weeks)	55% (asymptomatic reactivation)	100%

GVHD: Graft-versus-host disease; PPX: Prophylaxis; ORR: Overall response rate: CR: Complete response; CsA: Cyclosporine A; MTX: Methotrexate; MMF: Mycophenolate mofetil; CMV: Cytomegalovirus.

## References

[1] Thomas E., Storb R., Clift A.R., Fefer A., Johnson F.L., Neiman P.E., Lerner K.G., Glucksberg H., Buckner C.D. (1975). Bone-marrow transplantation (first of two parts). N. Engl. J. Med..

[2] Poire X., van Besien K. (2011). Alemtuzumab in allogeneic hematopoetic stem cell transplantation. Exp. Opin. Biol. Ther..

[3] Beatty P.G., Mori M., Milford E. (1995). Impact of racial genetic polymorphism on the probability of finding an HLA-matched donor. Transplantation.

[4] Beatty P.G., Ash R., Hows J.M., McGlave P.B. (1989). The use of unrelated bone marrow donors in the treatment of patients with chronic myelogenous leukemia: Experience of four marrow transplant centers. Bone Marrow Transplant..

[5] McGlave P.B., Beatty P., Ash A., Hows J.M. (1990). Therapy for chronic myelogenous leukemia with unrelated donor bone marrow transplantation: Results in 102 cases. Blood.

[6] Mackinnon S., Barnett L., Heller G., O’Reilly R.J. (1994). Minimal residual disease is more common in patients who have mixed T-cell chimerism after bone marrow transplantation for chronic myelogenous leukemia. Blood.

[7] McGlave P., Bartsch G., Anasetti A., Ash A., Beatty P., Gajewski J., Kernan N.A. (1993). Unrelated donor marrow transplantation therapy for chronic myelogenous leukemia: Initial experience of the National Marrow Donor Program. Blood.

[8] Marks D.I., Cullis J.O., Ward K.N., Lacey S., Szydlo R., Hughes T.P., Schwarer A.P., Lutz E., Barrett A.J., Hows J.M. (1993). Allogeneic bone marrow transplantation for chronic myeloid leukemia using sibling and volunteer unrelated donors. A comparison of complications in the first 2 years. Ann. Intern. Med..

[9] Gooley T.A., Chien J.W., Pergam S.A., Hingorani S., Sorror M.L., Boeckh M., Martin P.J., Sandmaier B.M., Marr K.A., Appelbaum F.R. (2010). Reduced mortality after allogeneic hematopoietic-cell transplantation. N. Engl. J. Med..

[10] Perez-Simon J.A., Kottaridis P.D., Martino R., Craddock C., Caballero D., Chopra R., García-Conde J., Milligan D.W., Schey S., Urbano-Ispizua A. (2002). Nonmyeloablative transplantation with or without alemtuzumab: Comparison between 2 prospective studies in patients with lymphoproliferative disorders. Blood.

[11] Willemsen L., Jol-van der Zijde C.M., Admiraal R., Putter H., Jansen-Hoogendijk A.M., Ostaijen-ten Dam M.M., Wijnen J.T., van Kesteren C., Waaijer J.L.M., Lankester A.C. (2015). Impact of serotherapy on immune reconstitution and survival outcomes after stem cell transplantations in children: Thymoglobulin versus alemtuzumab. Biol. Blood Marrow Transplant..

[12] Storek J. (2015). Impact of serotherapy on immune reconstitution and survival outcomes after stem cell transplantations in children: Thymoglobulin versus alemtuzumab. Biol. Blood Marrow Transplant..

[13] Mohty M. (2007). Mechanisms of action of antithymocyte globulin: T-cell depletion and beyond. Leukemia.

[14] Stauch D., Dernier A., Marchese E.S., Kunert K., Volk H.-D., Pratschke J., Kotsch K. (2009). Targeting of natural killer cells by rabbit antithymocyte globulin and campath-1H: Similar effects independent of specificity. PLoS ONE.

[15] Veys P., Wynn R.F., Ahn K.W., Samarasinghe S., He W., Bonney D., Craddock J., Cornish J., Davies S.M., Dvorak C.C. (2012). Impact of immune modulation with in vivo T-cell depletion and myleoablative total body irradiation conditioning on outcomes after unrelated donor transplantation for childhood acute lymphoblastic leukemia. Blood.

[16] Hoegh-Petersen M., Amin M.A., Liu Y., Ugarte-Torres A., Williamson T.S., Podgorny P.J., Russell J.A., Grigg A., Ritchie D., Storek J. (2013). Anti-thymocyte globulins capable of binding to T and B cells reduce graft-vs-host disease without increasing relapse. Bone Marrow Transplant..

[17] Jol-van der Zijde C.M., Bredius R.G.M., Jansen-Hoogendijk A.M., Raaijmakers S., Egeler R.M., Lankester A.C., van Tol M.J.D. (2012). IgG antibodies to ATG early after pediatric hematopoietic SCT increase the risk of acute GVHD. Bone Marrow Transplant..

[18] Shah A.J., Kapoor N., Crooks G.M., Weinberg K.I., Azim H.A., Killen R., Kuo L., Rushing T., Kohn D.B., Parkman R. (2007). The effects of Campath 1H upon graft-versus-host disease, infection, relapse, and immune reconstitution in recipients of pediatric unrelated transplants. Biol. Blood Marrow Transplant..

[19] Schmidt-Hieber M., Schwarck S., Stroux A., Ganepola S., Reinke P., Thiel E., Uharek L., Blau I.W. (2010). Immune reconstitution and cytomegalovirus infection after allogeneic stem cell transplantation: The important impact of in vivo T cell depletion. Int. J. Hematol..

[20] Waller E.K., Langston A.A., Lonial S., Cherry J., Somani J., Allen A.J., Rosenthal H., Redei I. (2003). Pharmacokinetics and pharmacodynamics of anti-thymocyte globulin in recipients of partially HLA-matched blood hematopoietic progenitor cell transplantation. Biol. Blood Marrow Transplant..

[21] Chakrabarti S., Mackinnon S., Chopra R., Kottaridis P.D., Peggs K., O’Gorman P., Chakraverty R., Marshall T., Osman H., Mahendra P. (2002). High incidence of cytomegalovirus infection after nonmyeloablative stem cell transplantation: Potential role of Campath-1H in delaying immune reconstitution. Blood.

[22] Waldmann H. (2002). A personal history of the CAMPATH-1H antibody. Med. Oncol..

[23] Hale G., Waldmann H. (1998). Risks of developing Epstein-Barr virus-related lymphoproliferative disorders after T-cell-depleted marrow transplants. Blood.

[24] Ram R., Storb R. (2013). Pharmacologic prophylaxis regimens for acute graft-versus-host disease: Past, present and future. Leuk. Lymphoma.

[25] Sullivan K.M., Agura E., Anasetti C., Appelbaum F., Badger C., Bearman S., Erickson K., Flowers M., Hansen J., Loughran T. (1991). Chronic graft-versus-host disease and other late complications of bone marrow transplantation. Semin. Hematol..

[26] Toubai T., Mathewson N.D., Magenau J., Reddy P. (2016). Danger Signals and Graft-versus-host Disease: Current Understanding and Future Perspectives. Front. Immunol..

[27] Ball L.M., Egeler R.M., Party E.P.W. (2008). Acute GvHD: Pathogenesis and classification. Bone Marrow Transplant..

[28] Coghill J.M., Sarantopoulos S., Moran T.P., Murphy W.J., Blazar B.R., Serody J.S. (2011). Effector CD4+ T cells, the cytokines they generate, and GVHD: Something old and something new. Blood.

[29] Jagasia M.H., Greinix H.T., Arora M., Williams K.M., Wolff D., Cowen E.W., Palmer J., Weisdorf D., Treister N.S., Cheng G.-S. (2015). National Institutes of Health Consensus Development Project on Criteria for Clinical Trials in Chronic Graft-versus-Host Disease: I. The 2014 Diagnosis and Staging Working Group report. Biol. Blood Marrow Transplant..

[30] Lee S.-E., Kim Y.-J., Kim H.-J., Lee S., Min C.-K., Cho S.-G., Kim D.-W., Lee J.-W., Min W.-S., Park C.-W. (2013). Risk and prognostic factors for acute GVHD based on NIH consensus criteria. Bone Marrow Transplant..

[31] Martin P.J., Rizzo J.D., Wingard J.R., Ballen K., Curtin P.T., Cutler C., Litzow M.R., Nieto Y., Savani B.N., Schriber J.R. (2012). First- and second-line systemic treatment of acute graft-versus-host disease: Recommendations of the American Society of Blood and Marrow Transplantation. Biol. Blood Marrow Transplant..

[32] Hings I.M., Filipovich A.H., Milier W.J., Blazar B.L., Mcalave P.B., Rmasay N.K.C., Kersey J.H., Weisdore D.J. (1993). Prednisone therapy for acute graft-versus-host disease: Short- versus long-term treatment. A prospective randomized trial. Transplantation.

[33] Van Lint M.T., Milone G., Leotta S., Uderzo C., Scimè R., Dallorso S., Locasciulli A., Guidi S., Mordini N., Sica S. (2006). Treatment of acute graft-versus-host disease with prednisolone: Significant survival advantage for day +5 responders and no advantage for nonresponders receiving anti-thymocyte globulin. Blood.

[34] MacMillan M.L., Milone G., Leotta S., Uderzo C., Scimè R., Dallorso S., Locasciulli A., Guidi S., Mordini N., Sica S. (2002). Response of 443 patients to steroids as primary therapy for acute graft-versus-host disease: Comparison of grading systems. Biol. Blood Marrow Transplant..

[35] Deeg H.J. (2007). How I treat refractory acute GVHD. Blood.

[36] Kawahara Y., Morimoto A., Masuzawa A., Ikeda T., Hayase T., Kashii Y., Nozaki Y., Kanai N., Momoi M.Y. (2012). Successful treatment with pulse cyclophosphamide of a steroid-refractory hepatitic variant of liver acute graft-vs.-host disease in a child. Pediatr. Transl..

[37] Mayer J., Krejci M., Pospisil Z., Doubek M., Janikova A., Zackova D., Racil Z., Smardova L., Navratil M., Kamelander J. (2009). Successful treatment of steroid-refractory hepatitic variant of liver graft-vs-host disease with pulse cyclophosphamide. Exp. Hematol..

[38] Byrne J.L., Stainer C., Cull C., Haynes A.P., Bessell E.M., Bessell G., Bessell H., Russell N.H. (2000). The effect of the serotherapy regimen used and the marrow cell dose received on rejection, graft-versus-host disease and outcome following unrelated donor bone marrow transplantation for leukaemia. Bone Marrow Transplant..

[39] Riechmann L., Clark M., Waldmann H., Winter G. (1988). Reshaping human antibodies for therapy. Nature.

[40] Sakellari I., Batsis I., Bousiou Z., Mallouri D., Constantinou V., Gavriilaki E., Smias C., Yannaki E., Kaloyannidis P., Papaioannou G. (2017). The Role of Low-dose Anti-thymocyte Globulin as Standard Prophylaxis in Mismatched and Matched Unrelated Hematopoietic Peripheral Stem Cell Transplantation for Hematologic Malignancies. Clin. Lymphoma Myeloma Leuk..

[41] Bunn D., Lea C.K., Bevan D.J., Higgins R.M., Hendry B.M. (1996). The pharmacokinetics of anti-thymocyte globulin (ATG) following intravenous infusion in man. Clin. Nephrol..

[42] Walker I., Panzarella T., Couban S., Couture F., Devins G., Elemary M., Gallagher G., Kerr H., Kuruvilla J., Lee S.J. (2016). Pretreatment with anti-thymocyte globulin versus no anti-thymocyte globulin in patients with haematological malignancies undergoing haemopoietic cell transplantation from unrelated donors: A randomised, controlled, open-label, phase 3, multicentre trial. Lancet Oncol..

[43] Hale G., Waldmann H. (1994). Control of graft-versus-host disease and graft rejection by T cell depletion of donor and recipient with Campath-1 antibodies. Results of matched sibling transplants for malignant diseases. Bone Marrow Transplant..

[44] Jacobs P., Wood L., Fullard L., Waldmann H., Hale G. (1994). T cell depletion by exposure to Campath-1G in vitro prevents graft-versus-host disease. Bone Marrow Transplant..

[45] Van der Zwan M., Baan C.C., van Gelder T., Hesselink D.A. (2017). Review of the Clinical Pharmacokinetics and Pharmacodynamics of Alemtuzumab and Its Use in Kidney Transplantation. Clin. Pharmacokinet..

[46] Hale G., Waldmann H. (1994). CAMPATH-1 monoclonal antibodies in bone marrow transplantation. J. Hematother..

[47] Watanabe T., Masuyama J., Sohma Y., Inazawa H., Horie K., Kojima K., Uemura Y., Aoki Y., Kaga S., Minota S. (2006). CD52 is a novel costimulatory molecule for induction of CD4+ regulatory T cells. Clin. Immunol..

[48] Page M.J., Sydenham M.A. (1991). High level expression of the humanized monoclonal antibody Campath-1H in Chinese hamster ovary cells. Biotechnology.

[49] Kanda J., Lopez R.D., Rizzieri D.A. (2011). Alemtuzumab for the prevention and treatment of graft-versus-host disease. Int. J. Hematol..

[50] Theurich S., Fischmann H., Shimabukuro-Vornhagen A., Chemnitz J.M., Holtick U., Scheid C., Skoetz N., von Bergwelt-Baildon M. (2012). Polyclonal anti-thymocyte globulins for the prophylaxis of graft-versus-host disease after allogeneic stem cell or bone marrow transplantation in adults. Cochrane Database Syst. Rev..

[51] Ziakas P.D., Zervou F.N., Zacharioudakis I.M., Mylonakis E. (2014). Graft-versus-host disease prophylaxis after transplantation: A network meta-analysis. PLoS ONE.

[52] Ayuk F., Diyachenko G., Zabelina T., Panse J., Wolschke C., Eiermann T., Binder T., Fehse B., Erttmann R., Kabisch H. (2008). Anti-thymocyte globulin overcomes the negative impact of HLA mismatching in transplantation from unrelated donors. Exp. Hematol..

[53] Friedrichs B., Tichelli A., Bacigalupo A., Russell N.H., Ruutu T., Shapira M.Y., Beksac M., Hasenclever D., Socié G., Schmitz N. (2010). Long-term outcome and late effects in patients transplanted with mobilised blood or bone marrow: A randomised trial. Lancet Oncol..

[54] Wolschke C., Zabelina T., Ayuk F., Alchalby H., Berger J., Klyuchnikov E., Pein U.-M., Schumacher S., Amtsfeld G., Adjallé R. (2014). Effective prevention of GVHD using in vivo T-cell depletion with anti-lymphocyte globulin in HLA-identical or -mismatched sibling peripheral blood stem cell transplantation. Bone Marrow Transplant..

[55] Socie G., Schmoor C., Bethge W.A., Ottinger H.D., Stelljes M., Zander A.R., Volin L., Ruutu T., Heim D.A., Schwerdtfeger R. (2011). Chronic graft-versus-host disease: Long-term results from a randomized trial on graft-versus-host disease prophylaxis with or without anti-T-cell globulin ATG-Fresenius. Blood.

[56] Kroger N., Solano C., Wolschke C., Bandini G., Patriarca F., Pini M., Nagler A., Selleri C., Risitano A., Messina G. (2016). Antilymphocyte Globulin for Prevention of Chronic Graft-versus-Host Disease. N. Engl. J. Med..

[57] Bryant A., Mallick R., Huebsch L.B., Allan D.S., Atkins H., Anstee G., Bence-Bruckler I., Hamelin L., Hodgins L., Sabloff M. (2017). Low-Dose Anti-Thymocyte Globulin for Graft-Versus-Host-Disease Prophylaxis in Matched Unrelated Allogeneic Hematopoietic Stem Cell Transplant. Biol. Blood Marrow Transplant..

[58] Tandra A., Mhaskar A.R., Kharfan-Dabaja M., Anasetti C., Mohty M., Djulbegovic B. (2016). Low Dose Antithymocyte Globulin (ATG) for Graft-Versus-Host Disease (GVHD) Prophylaxis. Blood.

[59] Crocchiolo R., Esterni B., Castagna L., Fürst S., El-Cheikh E., Devillier R., Granata A., Oudin C., Calmels B., Chabannon C. (2013). Two days of antithymocyte globulin are associated with a reduced incidence of acute and chronic graft-versus-host disease in reduced-intensity conditioning transplantation for hematologic diseases. Cancer.

[60] Wang Y., Fu H.-X., Liu D.-H., Xu L.-P., Zhang X.-H., Chang Y.-J., Chen Y.-H., Wang F.-R., Sun Y.-Q., Tang F.-F. (2014). Influence of two different doses of antithymocyte globulin in patients with standard-risk disease following haploidentical transplantation: A randomized trial. Bone Marrow Transplant..

[61] Bacigalupo A., Lamparelli T., Barisione G., Bruzzi P., Guidi S., Alessandrino P.E., di Bartolomeo P., Oneto R., Bruno B., Sacchi N. (2006). Thymoglobulin prevents chronic graft-versus-host disease, chronic lung dysfunction, and late transplant-related mortality: Long-term follow-up of a randomized trial in patients undergoing unrelated donor transplantation. Biol. Blood Marrow Transplant..

[62] Ruutu T., Gratwohl A., de Witte T., Afanasyev B., Apperley J., Bacigalupo A., Dazzi F., Dreger P., Duarte R., Finke J. (2014). Prophylaxis and treatment of GVHD: EBMT-ELN working group recommendations for a standardized practice. Bone Marrow Transplant..

[63] Admiraal R., van Kesteren C., Jol-van der Zijde C.M., Lankester A.C., Bierings M.B., Egberts T.C.G., van Tol M.J.D., Knibbe C.A.J., Bredius R.G.M., Boelens J.J. (2017). Association between anti-thymocyte globulin exposure and survival outcomes in adult unrelated haemopoietic cell transplantation: A multicentre, retrospective, pharmacodynamic cohort analysis. Lancet Haematol..

[64] Champlin R.E., Perez W.S., Passweg J.R., Klein J.P., Camitta B.M., Gluckman E., Bredeson C.N., Eapen M., Horowitz M.M. (2007). Bone marrow transplantation for severe aplastic anemia: A randomized controlled study of conditioning regimens. Blood.

[65] Doney K.C., Weiden P.L., Storb R., Thomas E.D. (1981). Failure of early administration of antithymocyte globulin to lessen graft-versus-host disease in human allogeneic marrow transplant recipients. Transplantation.

[66] Hagen P., Wagner J.E., DeFor T.E., Dolan M., Arora M., Warlick E., Weisdorf D., Brunstein C.G. (2014). The effect of equine antithymocyte globulin on the outcomes of reduced intensity conditioning for AML. Bone Marrow Transplant..

[67] Hale G., Zhang M.-J., Bunjes D., Prentice H.G., Spence D., Horowitz M.M., Barrett A.J., Waldmann H. (1998). Improving the outcome of bone marrow transplantation by using CD52 monoclonal antibodies to prevent graft-versus-host disease and graft rejection. Blood.

[68] Chakraverty R., Peggs K., Chopra R., Milligan D.W., Kottaridis D.P., Verfuerth S., Geary J., Thuraisundaram D., Branson K., Chakrabarti S. (2002). Limiting transplantation-related mortality following unrelated donor stem cell transplantation by using a nonmyeloablative conditioning regimen. Blood.

[69] Kottaridis P.D., Milligan D.W., Chopra R., Chakraverty R.K., Chakrabarti S., Robinson S., Peggs K., Verfuerth S., Pettengell R., Marsh J.C.W. (2000). In vivo CAMPATH-1H prevents graft-versus-host disease following nonmyeloablative stem cell transplantation. Blood.

[70] Van Besien K., Artz A., Smith S., Cao D., Rich S., Godley L., Jones D., Del Cerro P., Bennett D., Casey B. (2005). Fludarabine, melphalan, and alemtuzumab conditioning in adults with standard-risk advanced acute myeloid leukemia and myelodysplastic syndrome. J. Clin. Oncol..

[71] Van Besien K., Kunavakkam R., Rondon G., De Lima M., Artz A., Oran B., Giralt S. (2009). Fludarabine-melphalan conditioning for AML and MDS: Alemtuzumab reduces acute and chronic GVHD without affecting long-term outcomes. Biol. Blood Marrow Transplant..

[72] Malladi R.K., Peniket A.J., Littlewood T.J., Towlson K.E., Pearce R., Yin J., Cavenagh J.D., Craddock C., Orchard K.H., Olavarria E. (2009). Alemtuzumab markedly reduces chronic GVHD without affecting overall survival in reduced-intensity conditioning sibling allo-SCT for adults with AML. Bone Marrow Transplant..

[73] Freytes C.O., Loberiza F.R., Rizzo J.D., Bashey A., Bredeson C.N., Cairo M.S., Gale R.P., Horowitz M.M., Klumpp T.R., Martino R. (2004). Myeloablative allogeneic hematopoietic stem cell transplantation in patients who experience relapse after autologous stem cell transplantation for lymphoma: A report of the International Bone Marrow Transplant Registry. Blood.

[74] Morris E., Mackinnon S. (2005). Outcome following alemtuzumab (CAMPATH-1H)-containing reduced intensity allogeneic transplant regimen for relapsed and refractory non-Hodgkin’s lymphoma (NHL). Transfus. Apher. Sci..

[75] Morris E., Thomson K., Craddock C., Mahendra P., Milligan D., Cook G., Smith M.G., Parker A., Schey S., Chopra R. (2004). Outcomes after alemtuzumab-containing reduced-intensity allogeneic transplantation regimen for relapsed and refractory non-Hodgkin lymphoma. Blood.

[76] Thomson K.J., Morris E.C., Milligan D., Parker A.N., Hunter A.E., Cook G., Bloor A.J.C., Clark F., Kazmi M., Linch D.C. (2010). T-cell-depleted reduced-intensity transplantation followed by donor leukocyte infusions to promote graft-versus-lymphoma activity results in excellent long-term survival in patients with multiply relapsed follicular lymphoma. J. Clin. Oncol..

[77] Lim Z.Y., Ho A.Y.L., Ingram W., Kenyon M., Pearce L., Czepulkowski B., Devereux S., Duarte R.F., Pagliuca A., Mufti G.J. (2006). Outcomes of alemtuzumab-based reduced intensity conditioning stem cell transplantation using unrelated donors for myelodysplastic syndromes. Br. J. Haematol..

[78] Kline J., Pollyea D.A., Stock W., Artz A., Rich E., Godley L., Zimmerman T., Thompson K., Pursell K., Larson L.A. (2006). Pre-transplant ganciclovir and post transplant high-dose valacyclovir reduce CMV infections after alemtuzumab-based conditioning. Bone Marrow Transplant..

[79] Carpenter B., Haque T., Dimopoulou M., Atkinson C., Roughton M., Grace S., Denovan S., Fielding A., Kottaridis P., Griffiths P. (2010). Incidence and dynamics of Epstein-Barr virus reactivation after alemtuzumab-based conditioning for allogeneic hematopoietic stem-cell transplantation. Transplantation.

[80] Ho V.T., Soiffer R.J. (2001). The history and future of T-cell depletion as graft-versus-host disease prophylaxis for allogeneic hematopoietic stem cell transplantation. Blood.

[81] Storb R., Prentice R.L., Buckner C.D., Clift R.A., Appelbaum F., Deeg J., Doney K., Hansen K., Mason M., Sanders J.E. (1983). Graft-versus-host disease and survival in patients with aplastic anemia treated by marrow grafts from HLA-identical siblings. Beneficial effect of a protective environment. N. Engl. J. Med..

[82] Ringden O., Nilsson B. (1985). Death by graft-versus-host disease associated with HLA mismatch, high recipient age, low marrow cell dose, and splenectomy. Transplantation.

[83] Kanakry C.G., Fuchs E.J., Luznik L. (2016). Modern approaches to HLA-haploidentical blood or marrow transplantation. Nat. Rev. Clin. Oncol..

[84] Cragg L., Blazar B.R., Defor T., Kolatker N., Miller W., Kersey J., Ramsay N., McGlave P., Filipovich A., Weisdorf D. (2000). A randomized trial comparing prednisone with antithymocyte globulin/prednisone as an initial systemic therapy for moderately severe acute graft-versus-host disease. Biol. Blood Marrow Transplant..

[85] Remberger M., Aschan J., Barkholt L., Tollemar J., Ringdén O. (2001). Treatment of severe acute graft-versus-host disease with anti-thymocyte globulin. Clin. Transplant..

[86] Ozen M., Bozdag S., Cakmak G., Topcuoglu P., Eroglu A.H., Gunduz M., Arslan O., Demirer T., Akan H., Ilhan O. (2015). Antilymphocyte/Thymocyte Globulin for the Treatment of Steroid-Refractory Acute Graft-Versus-Host Disease: 20-Year Experience at a Single Center. Int. J. Hematol. Oncol..

[87] Khoury H., Kashyap A., Adkins D.R., Brown R.A., Miller G., Vij R., Westervelt P., Trinkaus K., Goodnough L.T., Hayashi R.J. (2001). Treatment of steroid-resistant acute graft-versus-host disease with anti-thymocyte globulin. Bone Marrow Transplant..

[88] Arai S., Margolis J., Zahurak M., Zahurak V., Vogelsang G.B. (2002). Poor outcome in steroid-refractory graft-versus-host disease with antithymocyte globulin treatment. Biol. Blood Marrow Transplant..

[89] McCaul K.G., Nevill T.J., Barnett M.J., Toze C.L., Currie C.J., Sutherland H.J., Conneally E.A., Shepherd J.D., Nantel S.H., Hogge D.E. (2000). Treatment of steroid-resistant acute graft-versus-host disease with rabbit antithymocyte globulin. J. Hematother. Stem Cell Res..

[90] MacMillan M.L., Weisdorf D.J., Davies S.M., DeFor T.E., Burns L.J., Burns N.K.C., Wagner J.E., Blazar B.R. (2002). Early antithymocyte globulin therapy improves survival in patients with steroid-resistant acute graft-versus-host disease. Biol. Blood Marrow Transplant..

[91] Hsu B., May R., Carrum G., Krance R., Przepiorka D. (2001). Use of antithymocyte globulin for treatment of steroid-refractory acute graft-versus-host disease: An international practice survey. Bone Marrow Transplant..

[92] Shlomchik W.D., Couzens M.S., Tang C.B., McNiff J., Robert M.E., Liu J., Shlomchik M.J., Emerson M.G. (1999). Prevention of graft versus host disease by inactivation of host antigen-presenting cells. Science.

[93] Matte C.C., Liu J., Cormier J., Anderson B.E., Athanasiadis I., Jain D., McNiff J., Shlomchik W.D. (2004). Donor APCs are required for maximal GVHD but not for GVL. Nat. Med..

[94] Anderson B.E., McNiff J.M., Jain D., Blazar B.R., Shlomchik W.D., Shlomchik M.J. (2005). Distinct roles for donor- and host-derived antigen-presenting cells and costimulatory molecules in murine chronic graft-versus-host disease: Requirements depend on target organ. Blood.

[95] Collin M.P., Munster Da., Clark G., Wang X.-N., Dickinson A.M., Hart D.N. (2005). In vitro depletion of tissue-derived dendritic cells by CMRF-44 antibody and alemtuzumab: Implications for the control of Graft-versus-host disease. Transplantation.

[96] Von dem Borne P.A., Beaumont F., Starrenburg C.W., Oudshoorn M., Hale G., Falkenburg J.H., Fibbe M.E., Willemze R., Barge R.M. (2006). Outcomes after myeloablative unrelated donor stem cell transplantation using both in vitro and in vivo T-cell depletion with alemtuzumab. Haematologica.

[97] Delgado J., Thomson K., Russell N., Ewing J., Stewart W., Cook G., Devereux S., Lovell R., Chopra R., Marks D.I. (2006). Results of alemtuzumab-based reduced-intensity allogeneic transplantation for chronic lymphocytic leukemia: A British Society of Blood and Marrow Transplantation Study. Blood.

[98] Gomez-Almaguer D., Ruiz-Argüelles G.J., Tarín-Arzaga L.D.C., González-Llano O., Gutiérrez-Aguirre H., Cantú-Rodríguez O., Jaime-Pérez J., Carrasco-Yalán A., Giralt S. (2008). Alemtuzumab for the treatment of steroid-refractory acute graft-versus-host disease. Biol. Blood Marrow Transplant..

[99] Martinez C., Solano C., Ferrá C., Sampol A., Valcárcel D., Pérez-Simón J.A. (2009). Spanish Group for Stem Cell Transplantation. Alemtuzumab as treatment of steroid-refractory acute graft-versus-host disease: Results of a phase II study. Biol. Blood Marrow Transplant..

[100] Schnitzler M., Hasskarl J., Egger M., Bertz H., Finke J. (2009). Successful treatment of severe acute intestinal graft-versus-host resistant to systemic and topical steroids with alemtuzumab. Biol. Blood Marrow Transplant..

[101] Schub N., Günther A., Schrauder A., Claviez A., Ehlert C., Gramatzki M., Repp R. (2011). Therapy of steroid-refractory acute GVHD with CD52 antibody alemtuzumab is effective. Bone Marrow Transplant..

[102] Ruiz-Arguelles G.J., Gil-Beristain J., Magaña M., Ruiz-Delgado G.J. (2008). Alemtuzumab-induced resolution of refractory cutaneous chronic graft-versus-host disease. Biol. Blood Marrow Transplant..

[103] Ruiz-Arguelles G.J., Ruiz-Delgado G.J., Moreno-Ford V. (2008). Re: Alemtuzumab-induced resolution of pulmonary noninfectious complications in a patient with chronic graft-versus-host disease. Biol. Blood Marrow Transplant..

